# Moving anticoagulation initiation and monitoring services into the community: evaluation of the Brighton and hove community pharmacy service

**DOI:** 10.1186/s12913-018-2901-8

**Published:** 2018-02-07

**Authors:** Samantha J. Ingram, Charlotte L. Kirkdale, Sian Williams, Elaine Hartley, Susan Wintle, Valerie Sefton, Tracey Thornley

**Affiliations:** 1 Boots UK Ltd, Thane Road, Beeston, Nottingham, UK; 2 School of Pharmacy & Biomolecular Science, University of Brighton, Brighton, UK; 3 School of Pharmacy, University of Nottingham, Nottingham, UK

**Keywords:** Anticoagulation, Commissioning, Community pharmacy, INR, Warfarin

## Abstract

**Background:**

As part of the NHS desire to move services closer to where people live, and provide greater accessibility and convenience to patients, Brighton and Hove Clinical Commissioning Group (CCG) underwent a review of their anticoagulation services during 2008. The outcome was to shift the initiation and monitoring service in secondary care for non-complex patients, including domiciliary patients, into the community. This was achieved via a procurement process in 2008 resulting in the Community Pharmacy Anticoagulation Management Service (CPAMs) managed by Boots UK (a large chain of community pharmacies across the United Kingdom).

**Methods:**

This evaluation aims to review the outcomes (International Normalised Ratio [INR] readings) and experiences of those patients attending the anticoagulation monitoring service provided by community pharmacists in Brighton and Hove. All patients on warfarin are given a target INR range they need to achieve; dosing of and frequency of appointment are dependent on the INR result. Outcome measures for patients on the CPAM service included percentage INR readings that were within target range and the percentage time the patient was within therapeutic range. Data collected from 2009 to 2016 were analysed and results compared to the service targets. Patient experience of the service was evaluated via a locally developed questionnaire that was issued to patients annually in the pharmacy.

**Results:**

The evaluation shows that community pharmacy managed anticoagulation services can achieve outcomes at a level consistently exceeding national and local targets for both percentage INR readings in therapeutic target range (65.4%) compared to the recommended minimum therapeutic target range of 60.0% and percentage time in therapeutic range (72.5%, CI 71.9–73.1%) compared to the national target of 70.0%. Patients also indicated they were satisfied with the service, with over 98.6% patients rating the service as good, very good or excellent.

**Conclusion:**

The Brighton and Hove CPAM service achieved above average national target management of INR and positive patient feedback, demonstrating that community pharmacy is ideally placed to provide this service safely and deliver enhanced clinical outcomes and positive patient experience.

**Electronic supplementary material:**

The online version of this article (10.1186/s12913-018-2901-8) contains supplementary material, which is available to authorized users.

## Background

Warfarin is the most commonly prescribed oral anticoagulant in the UK [1] and is used in the clinical management of a variety of conditions including atrial fibrillation (AF), post-myocardial infarction, heart valve replacement and deep vein thrombosis (DVT). A number of recognised side effects are associated with warfarin use, from nausea, vomiting and minor bleeding to more serious side effects such as major bleeding and warfarin-induced skin necrosis [2]. Such side effects have been identified as a causal agent in 10% of all admissions for adverse drug reactions [3].

Experience of side effects of warfarin use are related to a patient’s International Normalised Ratio (INR) level, a measure of the delay in blood clotting caused by warfarin. A supra-therapeutic INR can put patients at risk of bleeding, while prophylaxis against thromboembolic events may not be achieved with a sub-therapeutic range [4]. The specific recommended target range of INR values depends upon the disease and clinical condition. The aim of an anticoagulant monitoring service is to stabilise the INR within recommended targets for the clinical indication being managed while maximising effective treatment. These targets include percentage of readings that are within therapeutic range (RR) and the percentage time the patient is within therapeutic range (TTR).

The majority of anticoagulation monitoring currently takes place within secondary care (both hospital in- and out-patient settings). However, delivery of these services within secondary care faces a number of challenges which include an increase in numbers of older patients, clinical indications for warfarin and the accessibility of a centralised clinic setting supporting a wide geography. These challenges have been reiterated more generally in the National Health Service (NHS) 5 Year Forward View which highlights the efficiency savings the NHS must make and the push for moving more services from secondary care into the community [5]. The White Paper ‘Our Health, Our Care, Our Say’ (2006) highlighted how health and social care services needed to be closer to communities providing a flexible tailored service giving patients more control over their treatment [6]. Part of the English Government’s intent is to move clinical services, such as anticoagulation clinics, into the community to improve access and operational capacity within the local health economy [6]. Since the publication of these papers, more anticoagulation clinics are now within the community in places such as general practices (where a clinic may be run by a nurse or pharmacist), within community pharmacies, or at patient’s homes with the use of self-testing.

Other countries have already experienced some success with providing anticoagulation monitoring through community pharmacy, for example the Community Pharmacy-Based Anticoagulation Management Service in New Zealand [7] and other pilots in Canada and the US [8–10]. Whilst some pharmacy-run clinics have been in place since 1979 [11], there has been limited access to primary provision of anticoagulation in the UK until recent years. In recognition that this is a complex area for planning and commissioning, the National Institute for Health and Care Excellence (NICE) has developed a Commissioning Guide to support the NHS to commission high quality, evidence based anticoagulant services with the potential to change local care pathways [12]. This has impacted on where such services are provided; in secondary or community care.

The development of anticoagulation clinics led by pharmacists could significantly increase patient access to professional advice and testing, leading to improved INR control and self-care [13]. Pharmacists are well placed to take on the role of practitioner in this field as their knowledge of drug interactions, pharmaceutical products, pharmacokinetic principles and counselling skills enables them to effectively manage patients safely within agreed treatment protocols.

In 2008 Boots UK was awarded the NHS local contract to operate a community pharmacy led service in the Brighton and Hove area of England. The Community Pharmacy Anticoagulation Management (CPAM) service was implemented in 17 pharmacies (nine Boots UK, and eight independently owned pharmacies) across the locality in October 2009, with a mixture of pharmacies of different sizes and locations (e.g. local, high street, large city centre). The aim of this service evaluation was to compare outcomes achieved locally to recommended targets for percentage RR and percentage TTR, and assess levels of patient satisfaction.

## Methods

Boots UK has managed the CPAM service since 2009. Other pharmacy providers continue to be subcontracted to ensure good geographical coverage. ‘Here’, formally Brighton and Hove Integrated Care Services Ltd., was engaged to manage patient referral and escalation of clinical queries to general practitioners (GPs) with a special interest in anticoagulation treatment. The CPAM contract stipulated the provision of access to GPs with specialist interest to provide advice to the pharmacists through electronic communication via the DAWN® AC Anticoagulation software version seven (4S Information Systems Ltd., Cumbria, UK). The service was commissioned to transfer and maintain existing noncomplex patients prescribed warfarin in secondary care within Brighton and Hove to clinics run on the pharmacy premises or via a domiciliary service. Patients were eligible for referral if they had at least two out of their last four readings in target range, and did not fall into the exclusion criteria (under 16 years; newly diagnosed with venous thromboembolism (VTE); VTE patients still on low molecular weight heparin; pregnant; antiphsopholipid syndrome). Initially, patients in the maintenance phase of treatment were chosen by secondary care clinicians using their clinical judgement; most patients had AF and were deemed to be stable by these clinicians having already completed the initiation period (usually 6 weeks from the start of treatment), these were considered to be ‘noncomplex’ patients. Noncomplex patients are also able to attend a community clinic for initiation of warfarin treatment to agreed protocols on referral from their doctor. Competencies for the service are in line with Brighton and Sussex University Hospital NHS Trust guidelines which are also in line with national recommendations provided by the National Patient Safety Agency (NPSA) [14].

Within Boots, a team of seven pharmacists who had completed consultation skills training and were assessed by the clinical lead as being competent to clearly communicate with patients on dosing and other relevant clinical discussions, were recruited to provide the service across eight pharmacies. In addition, independent pharmacies each identified a pharmacist to provide the service. A Boots pharmacist independent prescriber (a pharmacist accredited by the General Pharmaceutical Council to prescribe autonomously for any condition within their clinical competence) with specialist interest in anticoagulation was appointed as Lead Clinical Pharmacist for the service. All pharmacists attended the National Centre for Anticoagulation Training at the University of Birmingham and completed a three day training course and final examination in oral anticoagulation management. A one-day local training course was also provided to all pharmacists covering service procedures, finger prick sampling technique, use of the point of care testing meter and the software systems. Each pharmacist observed the Lead Clinical Pharmacist treating ten patients, and was then observed themselves treating ten patients before final competency sign-off. Pharmacist competence was reviewed six-monthly via auditing of the service data and annually by observation of clinical practise by the Lead Clinical Pharmacist to ensure standards were maintained.

At each clinic appointment a patient’s INR was measured by taking a capillary blood sample using a finger prick device from the CoaguChek® XS Plus meter kit (Roche, Basel, Switzerland) and analysed using the same device. The INR result was input to an IT web based software solution, DAWN® AC Anticoagulation software, which provided pharmacists with support on dosing and recall interval decisions. The system also provides an appointment book which can track patients and ensure follow up appointments are audited and actioned, as well as providing the pharmacists with detailed patient anticoagulation history and warfarin dosing algorithms. The patient recall interval for appointments was based on previous work by Ryan et al. [15]. At the end of each appointment details of patient’s INR results and time and date of their next appointment were conveyed verbally to ensure patient understanding and recorded in the patient’s information book to act as a reminder (‘yellow book’ provided by the NPSA). The target INR range for each patient was specified by the referring clinician and double checked by the pharmacist against recommended targets for each indication.

Data on the percentage RR (total number of readings within range divided by total number of visits) and average TTR for the service were extracted from the DAWN® AC database from October 2009 to September 2016. The Rosendaal method was used to calculate the percentage TTR for each patient [16]. This method calculates INR-specific person time by incorporating the frequency of INR measurements and their actual values while assuming that changes between consecutive INR measurements are linear over time. Data analysis was undertaken on the maintenance period of treatment, this excluded readings taken in the 6 week initiation period (unless otherwise stated).

Level of patient satisfaction with the service was measured by undertaking an annual patient survey using a questionnaire comprised of 14 questions. This was developed locally with NHS Brighton and Hove CCG and was based on other current local service questionnaires. In 2013 the questionnaire was amended to include the NHS friends and family test question which are answered on a five-point Likert scale [17]. The survey was carried out annually over an eight-week period from 2011. Patients accessing the service in this time were given a questionnaire and freepost envelope to allow completion at a convenient time. Implied patient consent was taken by return of the questionnaire and all data were anonymised.

Data extracted from Dawn® AC Anticoagulation software were analysed using Windows Excel 2013. Data from completed questionnaires was analysed using SPSS Version 22.

## Results

**Fig. 1:**
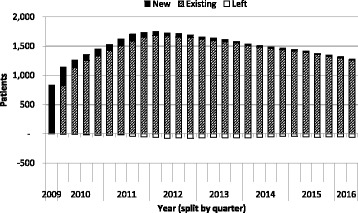
Turnover of patients over the course of the evaluation period. Bars show the loss of patients (‘left’) and the addition of ‘new’ patients by quarter

A total of 2341 patients were included in data analysis from October 2009 to June 2016. Due to the nature of this continuing service, patients were transferred out of the service (e.g. back to secondary care) or died, and new patients also joined the service throughout the evaluation period (see Fig. 1). Overall, a slightly higher proportion of patients were male (1293, 56.0%) and the majority of patients were white (2280, 97.6%; Table 1). Patients ranged from 22 to 106 years of age (as of September 2016 when data were extracted); most tending to be older, with 89.7% of patients aged 61 years or over (Table 1).

**Table 1:** Demographics of patients in the CPAM service (data were missing for some patients, percentages refer to known patients)

		Patients	
		Number	Percent
Gender (*n* = 2310)	Female	1017	44.0%
	Male	1293	56.0%
Ethnicity (*n* = 2337)	White	2280	97.6%
	Other	22	0.9%
	Asian	13	0.6%
	Black	9	0.4%
	Indian	6	0.3%
	Chinese	5	0.2%
	Bangladeshi	1	0.0%
	Pakistani	1	0.0%
Age group, in years (*n* = 2341)	21–30	12	0.5%
	31–40	27	1.2%
	41–50	68	2.9%
	51–60	134	5.7%
	61–70	332	14.2%
	71–80	615	26.3%
	81–90	879	37.5%
	≥91	274	11.7%

During the maintenance period (> 6 weeks treatment) a total of 168,316 INR readings were recorded over the evaluation period for 2341 patients. The average percentage RR for these patients, was 65.4% (Table 2). During the maintenance period, the average TTR for all patients was 72.5% (CI 71.9–73.1%, Table 2). The analysis demonstrate a slight decline in both metrics over the evaluation period (see Additional file 1: Figure A1). Any readings relating to the first quarter of a patient joining the service (including initiation period, ‘initial’) were compared with all readings which were not in the first quarter of joining (‘subsequent’). On average, the percentage TTR was higher for the ‘subsequent’ readings than the ‘initial’ readings (68.4% [CI 67.4–69.4] vs 37.8% [CI 32.2–43.4], *p* < 0.0001). This was also seen to be the case for percentage RR (64.4% ‘subsequent’ vs 46.8% for ‘initial’, *p* < 0.0001). A comparison was made between existing patients and those who left the service (defined as missing data in any subsequent quarterly period). This comparison suggests that patients who left the service had significantly lower readings for both metrics, than those who remained in the service (Table 2).

**Table 2:** Comparison of percentage TTR and RR for existing patients versus patients who have left the service

	Existing (*n* = 1169)	Left (*n* = 1172)	*P* value	All patients (*n* = 2341)
TTR % (CI)	78.0 (77.4–78.7%)	67.0 (66.1–68.0%)	*p* < 0.0001	72.5 (71.9–73.1%)
RR %	69.3%	61.4%	*p* < 0.0001	65.4

**Fig. 2:**
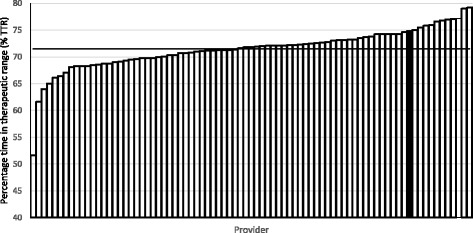
Comparative data between CPAM site (filled black column) and all other UK sites using DAWN®. Bars show the percentage TTR (obtained from DAWN® software providers) for the 6 months leading to April 2016 for each provider. The average TTR (71.5%) across all providers is shown as a solid horizontal line

The DAWN® software is also used by other providers of anticoagulation services across the UK (primary, community and secondary care), and comparison data were obtained from the software provider for all other users over a 6 month period leading to April 2016 (81 providers compared). Figure 2 shows that the Brighton and Hove CPAM service achieved above average outcomes for TTR and appears in the top 15 of providers.

A total of 2126 patients were surveyed about the service (401 patients after the first year [2011], 772 after the second year [2012] and 953 after the third year [2013]). The majority of respondents were male (56.4%), white (95.6%) and over 66 years old (80.2%). Overall satisfaction with the service was very high; this can be seen in Table 3.

**Table 3:** Frequency of patients’ overall ratings of the service (missing data, *n* = 43)

	Patients ratings	
Response	n	%
Very poor	2	0.1%
Poor	7	0.3%
Fair	21	1.0%
Good	112	5.4%
Very good	541	26.0%
Excellent	1400	67.2%
Total	2083	100%

Patient ratings of pharmacist interpersonal and communication skills were very high (Table 4). Ratings for information provided, however, were less positive (Table 4). In particular 13.0% (*n* = 267) of patients said that they wanted more information about why they were taking warfarin, 31.0% (*n* = 347) said they wanted more on side-effects and a further 2.8% (*n* = 31) patients said that they received no information about side-effects at all. The majority of patients found the pharmacy to be clean and tidy (85.4%) and the consultation room to be suitable (95.1%); 13.5% of patients reported some problems in contacting the pharmacy by phone.

**Table 4:** Percentage of positive ratings of pharmacists’ communication and interpersonal skills and information provided. Patient group size varies as some questions were not included in all 3 years of surveying patients and questions were non-compulsory

	Positive ratings by patients	
	n	%
Interpersonal and communication skills		
Listen carefully (*n* = 2060)	2048	99.4%
Time to discuss problems (*n* = 1937)	1930	99.6%
Answered questions^a^ (*n* = 1848)	1840	99.6%
Confidence and trust (*n* = 2087)	2086	99.9%
Respect and dignity (*n* = 2085)	2083	99.9%
Information		
Reason for taking warfarin (*n* = 2047)	1608	75.6%
Side-effects (*n* = 1119)	706	63.1%
Dose adjustment explained^a^ (*n* = 1780)	1772	99.6%
Use of the medicine (*n* = 1133)	933	82.3%
Referral to hospital - when & why^a^ (*n* = 183)	173	94.5%

^a^Where applicable

## Discussion

The evaluation has demonstrated that a community pharmacy managed anticoagulation service can achieve clinical outcomes at a level consistently exceeding targets; for percentage RR (65.4%) compared to the locally recommended minimum target of 60% and TTR (72.5%) compared to the national target of 70% [18, 19]. The initial patients transferred to the community service from the local NHS Trust were stable patients being prescribed warfarin for AF. As the number of patients transferred increased, so did the complexity of clinical conditions to further include initiation of warfarin treatment and those currently prescribed warfarin to manage DVT risk, pulmonary embolism risk, heart-valve disease and replacement heart valve management. It is already recognised that TTR tends to be lower in the first few months for newly warfarinised patients and our results support this. Coupled with the results, this suggests that patients joining the service tend to be less well maintained initially, this may explain the gradual reduction in the percentages reported for both measures although still consistently above NICE targets. This is reinforced by the DAWN® AC benchmarking data reporting the performance of all UK sites over a 6 month period including all sites within both primary and secondary care settings using the DAWN® AC dosing decision system. Although the patient populations may differ across these sites and so are not directly comparable, the benchmarking data suggest that the Brighton and Hove site is still performing well in comparison to other sites providing a similar service in UK. This evaluation supports previous studies which have shown that community pharmacy based anticoagulation clinics can be safe and clinically effective [20, 21].

Patients’ overall satisfaction for the service over the 3 years of data was very high, with feedback consistently rating above 98% at a minimum of ‘good’ whilst showing 67% rating the service as excellent. This mirrors other studies which have looked at community based clinical services [20, 21]. However, the findings show there is still an opportunity to provide more information to patients including discussing potential side effects which will continue to improve patient understanding and risk management of potential adverse events.

There are a number of limitations of this work, which impact the interpretation of the data. An example is the limited nature of the data collected. As the analysis is based on a simple audit, the reason for patients leaving the service and patient comorbidities and indications were not captured in the dataset. This means that it is not possible to comment on how many left due to death, treatment ending or switching to another treatment option or other such reasons; or whether there are any differences in warfarin management by indication. In future work, capturing these elements would provide valuable additional insights when evaluating the service. The survey data was collected anonymously which means that it is possible that the same patient may have responded to the survey in multiple years. Although this should be considered when interpreting the data, the patient’s overall rating of the service did not change greatly from year to year. In addition, the number of surveys handed out was not recorded, therefore unfortunately it is not possible to report on the response rate to the survey. As this was a simple audit and no data were available on patient co-morbidities etc., no analysis has been undertaken to account for risk-adjustment in the service population, or on the populations of the other providers, which should be taken into account when interpreting Fig. 2.

Anticoagulation management continues to evolve rapidly and developing expertise within this clinical area has also enabled the service to adapt in line with these changes. Whilst Any Qualified Provider (AQP) is a mechanism to commission such services, sole provider models are also popular, if the appropriate access to patients can be assured, allowing less contract management time for commissioners and an overview of the results of whole population outcomes. Further innovations on service delivery are currently being explored including piloting a model for patient self-testing; patients utilise their own CoaguChek Plus meters following an assessment by the anticoagulation pharmacist, and discuss results and dosing. Another potential proposal is the transition of the initiation clinic from a warfarin only service, to include a detailed discussion around the choice of a direct oral anticoagulant or warfarin in line with NICE clinical guidance CG180 [22]. Indeed, the slight decline in service users over the course of the analysis (Fig. 1), may be due to the increasing numbers of patients prescribed these new alternatives to warfarin treatment. This model utilises Independent Prescribers to initiate the first prescriptions, thereby reducing referral into secondary care. Both service innovations allow better access and choice for patients on anticoagulants and support patient empowerment, thus demonstrating that community pharmacy is able to adapt and meet the changing needs of patients.

## Conclusion

This evaluation has shown that community pharmacy is ideally positioned to support the commissioning of key services from primary care providing good clinical outcomes whilst attaining high patient satisfaction for the level of service and skills of the pharmacy team involved.

## Additional file

Additional file 1: Figure A1. Percentage TTR (A) and percentage of RR (B) by quarter (maintenance period) over the course of the service evaluation. (DOCX 24 kb)

## Abbreviations

AF: Atrial fibrillation
AQP: Any Qualified Provider
CCG: Clinical commissioning group
CPAM: Community Pharmacy Anticoagulation Management Service
DVT: Deep vein thrombosis
GP: General practitioner
INR: International Normalised Ratio
NHS: National Health Service
NICE: National Institute for Health and Care Excellence
NPSA: National Patient Safety Agency
TTR: Time in therapeutic range
UK: United Kingdom
